# Interventions Promoting Colorectal Cancer Screening Among Latino Men: A Systematic Review

**DOI:** 10.5888/pcd15.170218

**Published:** 2018-03-08

**Authors:** Cynthia M. Mojica, Deborah Parra-Medina, Sally Vernon

**Affiliations:** 1 School of Social and Behavioral Health Sciences, College of Public Health and Human Sciences, Oregon State University, Corvallis, Oregon; 2 Mexican American and Latina/o Studies, College of Liberal Arts, University of Texas at Austin, Austin, Texas; 3 Department of Health Promotion and Behavioral Sciences, University of Texas School of Public Health, Houston, Texas

## Abstract

**Introduction:**

Colorectal cancer, the second leading cause of cancer death in the United States, is also among the most preventable cancers. However, Latino men are less likely than non-Latino men to engage in preventive screening. Compared with 60% of non-Latino white men and women, only 42% of Latino men are up to date with colorectal cancer screening guidelines, which may result in diagnosis at advanced disease stages and increased deaths. We evaluated the literature on colorectal cancer screening interventions among Latino men to characterize intervention components effective in increasing colorectal cancer screening.

**Methods:**

Two independent reviewers searched MEDLINE, CINAHL, and PsycINFO to identify articles on intervention studies that promote colorectal cancer screening among Latino men. Inclusion criteria were randomized controlled or comparative effectiveness trials, an outcome of any colorectal cancer screening test, published in English, US-based, results published from January 2004 through December 2016, Latino or Spanish-speaking male participants, and a minimum of one patient-level component. Two other reviewers independently assessed article quality and conducted data abstraction.

**Results:**

Forty-four studies met the inclusion criteria; only 7 studies with 20% or more Latinos and 39% or more men were included in the final analyses. The most common intervention strategies included one-on-one interactions with a patient navigator and reducing structural barriers (eg, providing fecal occult blood tests). Interventions using small media produced mixed results.

**Conclusion:**

Although intervention studies focused on colorectal cancer screening among men of racial/ethnic minorities are scarce, our findings highlight promising strategies that were effective at increasing colorectal cancer screening among Latino men. Additional research in the area of Latino men’s health is needed, especially to further develop and test theoretically grounded interventions that promote colorectal cancer screening with larger samples of men and across diverse geographic areas in the United States.

## Introduction

Despite being among the most preventable diseases, colorectal cancer is the second leading cause of cancer-related death among men and women in the United States. Every year, approximately 50,000 people in the Unites States die from the disease. Estimates indicate that 60% of deaths from colorectal cancer among men and women aged 50 or older could be prevented by early detection (1). The United States Preventive Services Task Force recommends that people aged 50 to 75 at average risk for colorectal cancer be screened with the fecal occult blood test (FOBT) every year, sigmoidoscopy every 5 years (with high-sensitivity FOBT every 3 years), or colonoscopy every 10 years. The Healthy People 2020 benchmark is for 70.5% of adults aged 50 to 75 to be screened for colorectal cancer according to the most recent national guidelines; yet, only 42% of Latino men and 47.5% of Latino women are up to date with screening compared with 60% of non-Latino white men and women (2). Consequently, Latino men and women are more likely than non-Hispanic whites to be diagnosed at advanced stages of the disease, even after accounting for differences in age and socioeconomic status, setting the stage for poorer survival rates.

Although mortality rates for colorectal cancer have decreased nationally for both men and women (3) in all racial/ethnic groups except American Indian/Alaska Natives, whose rates have remained stable, there has not been a similar decrease among Latino men (4). Whereas the mortality rate for white men has decreased by 3.0% per year, the rate for Hispanic men has decreased by only 1.5% per year (5,6). The low screening rates among Latinos may in part explain why colorectal cancer mortality rates for Latino men have not decreased as they have for white men (4). Latino men need effective strategies aimed at increasing colorectal cancer screening rates. Reasons for low screening rates range from socioeconomic to cultural to health system barriers (7–11). Effective interventions are needed to improve cancer screening rates among minority populations. However, there is little information on effective interventions for increasing colorectal cancer screening rates among Latino men. The objective of this systematic review was to identify components of effective interventions that have increased colorectal cancer screening among Latino men.

## Methods

### Data sources

We conducted an electronic search of MEDLINE, CINAHL, and PsychINFO to identify journal articles published from 2004 through 2016 that reported on intervention studies promoting colorectal cancer screening. Search terms used were the following combinations of Medical Subject Heading and keyword terms: *colorectal neoplasm, colorectal cancer, colon cancer, early detection of cancer, health education, health behavior, health promotion, intervention studies, prevention and control, randomized controlled trials, colon cancer screening, population screening, screening tests, screening intervention, and preventive health services*. We consulted with a health sciences librarian and adhered to the standards for systematic reviews of the Institute of Medicine (12) and the PRISMA Statement (13).

### Study selection

Articles included in the review met the following inclusion criteria: they discussed a randomized controlled or comparative effectiveness trial, the outcome was any colorectal cancer screening (ie, FOBT, sigmoidoscopy, or colonoscopy), the article was written in English, the trial was US-based, the article was published from January 2004 through December 2016, trial participants were Latino or Spanish-speaking men, and the trial had a minimum of one patient-level component. We identified 1,146 articles for review (974 through database searching and 172 through other sources, including similar reviews of colorectal cancer screening interventions (14–17) and scanning reference lists of articles meeting the inclusion criteria)(Figure).

**Figure:**
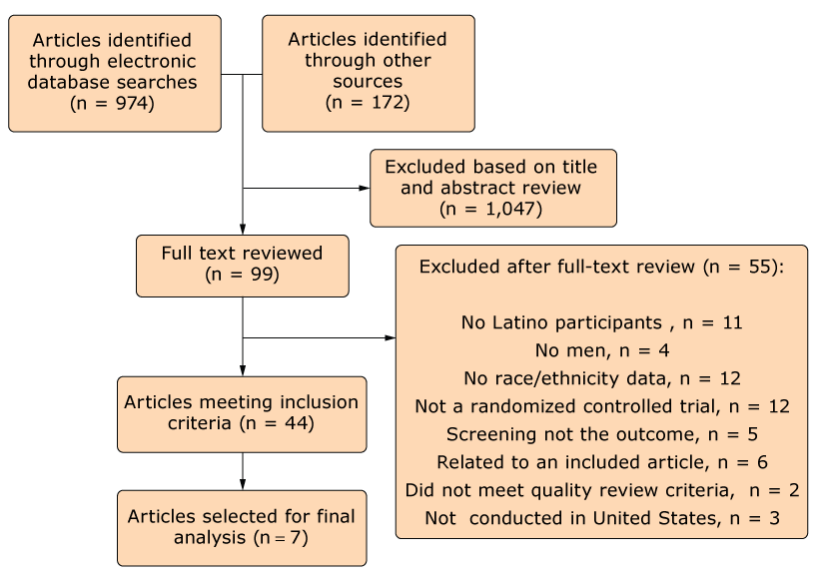
Flowchart showing inclusion process of articles analyzed in a systematic review of colorectal cancer screening among Latino men, United States, January 2004–December 2016.

Overall, the study selection process resulted in 44 articles meeting our inclusion criteria. However, the final sample selected for analysis consisted of 7 articles. We wanted to ensure that any conclusions based on this review were from articles with representative samples of Latinos and men. Thus, we looked at the distribution of Latinos and men in the study samples of the 44 articles and chose a cut-off at the median sample size, resulting in 7 articles with 20% or more Latinos and 39% or more men.

### Data extraction

We developed a data abstraction form to collect the following information: study year, title, study authors, participants (sample size, sex, age, and race/ethnicity), study design and setting, theoretical framework, intervention, screening outcome, and study results. C.M.M. extracted the data from each article that met the inclusion criteria, and D.P.M. evaluated the extracted data for accuracy. The 2 reviewers resolved disagreements by discussion. Our strategy meets the minimum recommended by the Centre for Reviews and Dissemination: one reviewer extracts the data while the second reviewer independently checks for accuracy and completeness of the extracted data (18).

Additionally, 2 other reviewers independently assessed the quality of each selected article using the Joanna Briggs Institute Critical Appraisal Checklist for Experimental Studies (19). This checklist for experimental studies examines 11 aspects of the article, including randomization, blinding of participants and staff, outcome assessment, follow up of participants, and appropriateness of statistical analyses. C.M.M. resolved any differences in quality ratings between the 2 reviewers.

## Results

### Search results

We summarized studies to describe the following characteristics for each of the 7 studies: study authors, study setting, sample (size, race/ethnicity, proportion of men), screening outcome (definition and measurement), intervention, and results (Table). All studies tested a patient-level intervention, and 5 were conducted in clinical settings (community health centers or Federally Qualified Health Centers). Two of the 7 studies were conducted in the Northeast (New York and Massachusetts), 1 in the Midwest (Illinois), 1 in the South (Texas), 2 in the West (Washington and California), and 1 study had a site in each region of the country (California, Colorado, Texas, and New York). Only 2 studies reported using a theoretical framework to develop the intervention. Except for 1 study that measured FOBT only, all studies used any colorectal cancer screening as the outcome measure. Five studies collected their outcome data via medical records. Almost all studies (n = 5) enrolled underserved populations. In Aragones et al (20), Coronado et al (21), and Jean-Jacques et al (22), 80%, 75%, and 73% of the sample, respectively, were uninsured or on public insurance. Enard et al enrolled Medicare enrollees, and 65% had less than a high school education (23). Percac-Lima et al enrolled a low-income population of which 46% were uninsured or on public insurance; however, no income information was reported (24). In contrast, 61% in Bastani et al (25) had some college education, and 63% of the population in Jerant et al (26) had a high school education or more.

**Table:** Summary of Studies (N =7), Interventions Promoting Colorectal Cancer (CRC) Screening Among Latino Men, United States, 2004–2016

Study Authors	Study Setting	Sample			Screening outcome	Intervention	Results
		Size	Race/Ethnicity	Men, n (%)			
Percac-Lima et al (24)	Massachusetts (urban community health center caring for a low-income population)	1,223	491 (40%) Latino, 67 (6%) African-American, 578 (47%) white, 28 (2%) Asian, 59 (5%) other	489 (40)	Any CRC screening (FOBT, colonoscopy, sigmoidoscopy, barium enema)^a^	Culturally tailored navigation vs usual care	Significant difference in screening between intervention group (27.4%) and usual care (11.9%) group
Aragones et al (20)	New York City (primary care clinic in teaching hospital caring for an underserved population)	65	Latino immigrants	31(48)	Any CRC screening^a^	11-Min video on portable personal digital video device plus brochure and 1-page reminder for physician vs usual care	Significant difference in screening between intervention group (55%) and usual care (18%) group
Coronado et al (21)	Washington State (community clinic caring for an underserved population)	501	Latino	235 (47)	FOBT^a^	Mailed FOBT with instructions vs mailed FOBT with instructions plus CHW education and home visits vs usual care	Significant difference in screening between intervention and usual care groups: 26% FOBT card with pamphlet vs 31% FOBT card with pamphlet plus *promotora*-education vs 2% usual care
Jean-Jacques et al (22)	Chicago (Federally Qualified Health Center caring for a low-income population)	202	41 (20%) Latino, 55 (27%) African-American, 53 (26%) white, 28 (14%) Asian, 25 (12%) other	125 (62)	FOBT, sigmoidoscopy, colonoscopy^a^	Outreach intervention (letter, fact sheet, mailed FOBT with instructions, telephone outreach by lay health educator) vs usual care	Significant difference in screening between intervention group (30%) and usual care (5%) group
Jerant et al (26)	Sacramento, CA; Bronx, NY; Rochester, NY; Colorado (Federally Qualified Health Centers and university-affiliated and private practices)	1,164	589 (51%) Latino, 279 (24%) African-American, 243 (21%) white, 53 (4%) other	767 (66)	FOBT, sigmoidoscopy, colonoscopy^a^	Tailored, interactive, multi-media computer program vs nontailored computer program^b^	No difference in screening between intervention group (23%) and control group (22%)
Bastani et al (25)	California (community-wide)	1,280	403 (32%) Latino, 284 (22%) African-American, 351 (27%) white, 242 (19%) Asian	564 (44)	Any CRC screening (FOBT, sigmoidoscopy, colonoscopy)^c^	Tailored print intervention plus barriers counseling vs usual care^b^, Group1: print intervention (6 mos), Group2: print intervention plus telephone call (12 mos)	Significant difference in screening between Group1 (15%) vs usual care (10%) and Group 2 (26%) vs usual care (18%)
Enard et al (23)	Texas (community-wide study targeting Medicare enrollees)	303	Latino	137 (45.2)	Any CRC screening (FOBT, sigmoidoscopy, colonoscopy)^b^	Tailored patient navigation vs control group (mailed educational materials describing general guidelines on CRC screening and other preventive services)	Significant difference in screening between intervention group (43.7%) and control (32.1%) group

Abbreviations: CHW, community health worker; FOBT, fecal occult blood text.

a Outcome was collected from medical records.

b A theoretical framework guided intervention development.

c Outcome was self-reported.

Intervention strategies used in these studies included 1) one-on-one interaction (eg, intensive in-person or phone contact with patients by a nurse, community health worker, or health educator) as the only component (n = 3) or combined with eliminating structural barriers (eg, providing an FOBT) (n = 2), and 2) incorporating small media with decision aids (materials that provide information on risks and benefits of screening and screening options) (n = 2). Six of 7 studies reported a significant difference in screening rate between the intervention and control groups. Personnel providing the one-on-one interaction were lay or college-educated community health workers (CHWs).

## Discussion

Findings from the 7 studies included in this review highlight the importance of using culturally appropriate strategies when targeting Latino men for colorectal cancer screening. The most common strategy was using patient navigation to promote colorectal cancer screening. Four studies used patient navigators (both those with and without a college education) and all significantly increased screening rates in the intervention group. Indeed, patient education combined with the use of patient navigators is an effective intervention strategy to increase colorectal cancer screening in minority (14) and Latino (17) populations. In this review, patient navigators, in their one-on-one encounters with patients, stressed the importance of cancer screening, explored and addressed barriers to screening, and provided opportunities for Latino men to ask questions. Two studies used lay CHWs who served as a link between the community and the health care system, assisting with outreach, health education, appointment scheduling, informal counseling, social support, and advocacy (27–31). Findings from this review further support the notion that CHWs can help increase colorectal cancer screening among Latino men. Along with the use of CHWs, both Coronado et al (21) and Jean-Jacques et al (22) provided mailed FOBTs, a method for reducing structural barriers, which in these studies was limiting the number of required clinic visits. These results are consistent with the *Community Guide*, which recommends one-on-one education and reducing structural barriers to increase colorectal cancer screening with FOBT (32). Results from Percac-Lima et al (24) and Enard et al (23) are inconsistent with the *Community Guide*, which does not recommend using these strategies to increase colorectal cancer screening with tests other than an FOBT. Given the results of Percac-Lima et al (24) and Enard et al (23), it may be that among Latino men (regardless of type of colorectal cancer screening test offered), patient navigation alone is an effective strategy, especially when navigation is tailored to individual needs and there is help navigating structural barriers. This possibility is further supported by Bastani et al (25) who delivered an ethnically targeted and tailored stepped, nested intervention (mail plus telephone call and no mailed FOBT) and reported that the intervention was effective among Latinos.

The second most common strategy was small media. Aragones et al (20) reported a significant increase in colorectal cancer screening that used a Spanish-language educational video that they evaluated by using pre-test and post-test surveys and focus group discussions. However, along with the video, participants also received a Spanish-language brochure and a one-page handout for their provider, making it difficult to know the independent effect of the video. Although there was no discussion of whether the video addressed cultural factors, the National Alliance for Hispanic Health developed the video in collaboration with community partners throughout the nation. Interestingly, the video was shown in Spanish despite 90% of participants in the intervention group reporting English as their primary language (only 35% said they spoke Spanish well to very well) (20). Jerant et al (26) also used small media that tailored the intervention on stage of readiness and theoretical factors associated with screening behavior. They did not find a significant increase in screening. The authors acknowledged that their findings might be due in part to not having addressed cultural factors affecting their Latino participants (26). Although we found only 2 studies testing small media, results were mixed and inconsistent with the *Community Guide*, which does not recommend small media to increase colorectal cancer screening by any other test except FOBT (32). In both of these studies, the interventions were designed to increase *any* screening — FOBT, sigmoidoscopy, or colonoscopy. Even so, the significant results of the Spanish-language video indicate the importance of tailoring materials and small media to language and creating products that are developed and evaluated with community input.

Our findings also highlight some important research gaps. First, culture, per se, was not explicitly addressed in these studies. The cultural tailoring referred to in the studies was mainly in regard to addressing patient language (providing materials in English and Spanish) and not cultural norms and beliefs. Only Percac-Lima et al (24) stated that they addressed cultural barriers; however, there were no details on what cultural barriers the patient navigators discussed with participants. Similarly, Bastani et al (25) targeted race/ethnicity only through culturally relevant photographs and graphics in the intervention materials. Second, except for Coronado et al (21), study authors did not report CHW sex. Although Coronado et al (21) reported hiring a male CHW (who was accompanied by a female medical assistant), there was no discussion of how CHW sex affected study findings. Yet, a recent review found that sex of the CHW, at least for women, is important in ensuring uptake of services, especially in low- and middle-income countries and when dealing with issues of maternal and child health (33,34). Similarly, in a study of diabetes management in the United States, the sex of the CHW mattered — for example, women may have to speak about sexual dysfunction associated with diabetes and they are uncomfortable doing so with male CHWs (35). Although a recent review of CHWs in Federally Qualified Health Centers described the characteristics of CHWs, such as training and education, they did not examine sex (36). Given the nature of colorectal cancer testing and the gender norms that characterize male Latino culture, it may be beneficial to examine whether male versus female CHWs are more effective in increasing colorectal cancer screening among Latino men. Third, only 2 studies reported using a theoretical framework to guide the planning, development, and evaluation of their intervention (25,26). Yet, research shows that interventions based on theory produce more effective and sustained behavior change than interventions designed without a theoretical framework (37). Also, without a theoretical framework, it is difficult to understand which components of an intervention, if any, were effective or why. Even so, a strength of these studies was the use of medical records to capture outcome data. However, given that the *Community Guide* recommends interventions based on colorectal cancer screening method, it would be helpful if studies, in addition to coding their outcome as *any* screening (ie, FOBT, sigmoidoscopy, or colonoscopy) also report the outcome by screening method.

Our study has limitations. First, our findings are subject to publication bias, because studies with negative findings are less likely to be published. Also, it is difficult to tease out which component of a multicomponent intervention was most effective. Lastly, although we carefully reviewed the literature, we may have missed some articles.

Despite these limitations, this review highlights the importance of interpersonal communication and tailoring to language and the need to address cultural norms. However, given that we found only 7 studies with a sufficient number of Latinos and men in the study samples, more research that investigates rates of colorectal cancer screening and interventions that promote screening among Latino men is warranted. This is even more important given that cancer has now surpassed heart disease as the number one cause of death among Latinos (2). Also, more research is needed to address the lack of studies testing the effectiveness of matching community health workers and their patients on sex. Lastly, 5 of the 7 studies in this review were conducted in a clinical setting. It is equally important to understand how to reach out to men in community settings. Research shows that men are not as connected to the health care system as women. From 2013 to 2015, 68% of low-income and uninsured men compared with 81% of low-income and uninsured women reported having a regular clinic they go to when sick or to ask for medical advice. Furthermore, only 75% of men reported seeing a provider in the past 2 years compared with 91% of women (38).

Overall, our findings highlight promising strategies — one-on-one education, use of small media, and reducing structural barriers — that may be effective in increasing colorectal cancer screening among Latino men. However, there is a clear need to continue doing theory-based intervention research with Latino men that explicitly addresses cultural beliefs and norms, beyond language.
